# Nabilone for the treatment of medication overuse headache: results of a preliminary double-blind, active-controlled, randomized trial

**DOI:** 10.1007/s10194-012-0490-1

**Published:** 2012-10-16

**Authors:** Luigi Alberto Pini, Simona Guerzoni, Maria Michela Cainazzo, Anna Ferrari, Paola Sarchielli, Ilaria Tiraferri, Michela Ciccarese, Maurizio Zappaterra

**Affiliations:** 1 Headache and Drug Abuse Interdepartmental Research Centre, University of Modena, Via del Pozzo 71, Modena, Italy; 2 Neurologic Clinic, Headache Centre, University of Perugia, Perugia, Italy

**Keywords:** Medication overuse headache (MOH), Treatment, Nabilone, Cannabinoid, Migraine

## Abstract

Medication overuse headache (MOH) is a severe burden to sufferers and its treatment has few evidence-based indications. The aim of this study is to evaluate efficacy and safety of nabilone in reducing pain and frequency of headache, the number of analgesic intake and in increasing the quality of life on patients with long-standing intractable MOH. Thirty MOH patients were enrolled at the University of Modena’s Interdepartmental Centre for Research on Headache and Drug Abuse (Italy) in a randomized, double-blind, active-controlled, crossover study comparing nabilone 0.5 mg/day and ibuprofen 400 mg. The patients received each treatment orally for 8 weeks (before nabilone and then ibuprofen or vice versa), with 1 week wash-out between them. Randomization and allocation (ratio 1:1) were carried out by an independent pharmacy through a central computer system. Participants, care givers, and those assessing the outcomes were blinded to treatment sequence. Twenty-six subjects completed the study. Improvements from baseline were observed with both treatments. However, nabilone was more effective than ibuprofen in reducing pain intensity and daily analgesic intake (*p* < 0.05); moreover, nabilone was the only drug able to reduce the level of medication dependence (−41 %, *p* < 0.01) and to improve the quality of life (*p* < 0.05). Side effects were uncommon, mild and disappeared when nabilone was discontinued. This is the first randomized controlled trial demonstrating the benefits of nabilone on headache, analgesic consumption and the quality of life in patients with intractable MOH. This drug also appears to be safe and well-tolerated. Larger scale studies are needed to confirm these preliminary findings.

## Introduction

Medication overuse headache (MOH) is a chronic headache (≥15 days/month) that develops from primary headaches (migraine, tension-type headaches). It has been described as the result of an interaction between an overused therapeutic agent and a susceptible patient [1, 2].

MOH is a common problem in tertiary headache centers, especially in patients with chronic migraine. The diagnosis is very important because patients seldom respond to prophylactic treatment, if the medication overuse for the acute condition continues [3–5].

MOH is a considerable burden for sufferers; its pathophysiology is unclear and its treatment has few evidence-based indications [1, 6, 7].

It has been suggested that this condition may be mediated by cognitive impulsiveness and has certain mechanisms in common with addiction and substance abuse [8].

A high percentage of patients with chronic daily headache with a high risk to develop MOH met the criteria for substance abuse according to the Diagnostic and Statistical Manual of Mental Disorders, Fourth Edition (DSM-IV). They also demonstrated that the prevalence of dependence according to DSM-IV varied with the different types of analgesic being overused [9].

In most cases, treatment of MOH includes an abrupt interruption of medication overuse through appropriate supportive care and the introduction of prophylactic treatment. There is no evidence on the most efficacious way to discontinue medication overuse. As the number of patients with this kind of problem continues to grow, MOH has become one of the main challenges of headache treatment in headache clinics [10–12].

Researches and current models are based on the assumption that it is caused by alterations in the nociceptive threshold and central sensitisation in susceptible individuals [13, 14].

These processes have a number of characteristics in common with chronic neuropathic pain or fibromyalgia, chronic conditions for which nabilone has been tested with encouraging results [15–17]. Increasing evidences are available concerning the benefits of cannabinoid agents in pain management, it should prompt to design larger and longer-term studies on their effects in homogeneous populations with chronic pain [18].

In one recent review of published studies on non-cancer pain, cannabinoids appeared to have proven safety and modest efficacy in the treatment of neuropathic pain whereas, there are some evidences of efficacy also in fibromyalgia and rheumatoid arthritis [19]. Other studies gave similar results in the management of neuropathic pain: one comparing nabilone and gabapentin used as add-on or mono-therapy in patients with peripheral neuropathy [20] and another comparing nabilone with dihydrocodeine in neuropathic pain [21].

Nabilone is a synthetic cannabinoid CB1-receptor agonist (licensed in Canada since 1981 for chemotherapy-induced vomiting and nausea); it is well-tolerated and has a good safety profile [22, 23]. Reports of its abuse are extremely rare and the drug has been even recently suggested to be a potential treatment for marijuana addiction [24, 25].

Cannabis derivatives have been suggested for the treatment of chronic pain conditions. Therefore, we tested the effects of nabilone in patients suffering from intractable/refractory MOH [26]. The study was aimed to investigate the efficacy of nabilone in reducing headache days, intensity of pain and analgesic intake in these patients.

The enrolled patients in the past performed many therapeutic attempt to withdraw daily analgesic abuse, without any clinical benefits.

It was well known by clinicians that their refractory headache patients did not suspend their antimigraine drugs without an alternative treatment.

Our ethic commitee did not allow to deprive patients suffering from daily headache of analgesic drugs by using a placebo, so we choose to treat daily attacks with a unique drug for all patients, by using ibuprofene as rescue medication or another drug if it was ineffective.

## Materials and methods

### Patients

Between February 2009 and May 2010, 30 outpatients attending the University of Modena and Reggio Emilia’s Interdepartmental Centre for Research on Headache and Drug Abuse (Italy) were enrolled.

Eligible patients were men and women who were not pregnant, aged between 35 and 65 years, with daily analgesic intake and who had MOH for at least 5 years. The age of headache onset had to be under 50 years and patients had to have already attempted detoxification at least three times, without success.

The diagnosis was formulated according to the ICHD-II criteria for MOH [27].

The exclusion criteria, at the screening visit, included blood test alterations and the previous continuative use of ibuprofen as anti-headache drug; systolic blood pressure >160 mmHg or diastolic pressure >100 mmHg; heart rate >100 bpm; patients with a history of drug addiction; patients with hypersensitivity to cannabinoids; patients not in possession of their full mental capacity or who have been declared legally incapacitated; patients with psychotic disorders or schizophrenia, bleeding disorders, pancreatic diseases, stomach or duodenal disorders, liver diseases, kidney diseases; patients treated with anticoagulants or antiplatelet agents and pregnant or breastfeeding women.

The study protocol was approved by the Independent Ethics Committee of Modena. The study was conducted in compliance with the provisions set forth in the Declaration of Helsinki (last version) and EU standards of Good Clinical Practice. All patients gave their written informed consent.

### Study design

A not-for-profit, independent, randomized, double-blind, active-controlled, crossover study (using a two period design, allocation ratio 1:1) was conducted on 30 outpatients attending the University of Modena’s Interdepartmental Centre for Research on Headache and Drug Abuse (Italy), where study visits took place, clinical data were collected and drugs were dispensed.

For treating headache attacks we decided to use a drug not overused by any patient, as safe as possible and that was not evaluable as potential prophylaptic treatment for headache.

Patients were randomly assigned to receive both treatments at home: one period with nabilone and one period with ibuprofen, in a blinded sequence. The drugs were taken orally every day and each treatment period lasted 8 weeks. Neither the doctors and nurses nor study patients knew which treatment sequence had been allocated.

Sixty doses of nabilone (0.5 mg) and 60 doses of ibuprofen (400 mg) were prepared for each patient by an independent pharmacy, as identical white capsules and randomized in two containers, named Drug A and Drug B. The pairs of containers were consecutively numbered for each subject according to the randomization schedule, generated by a computer. Each patient was assigned an order number and received the capsules in the corresponding pair of containers.

**Fig. 1:**
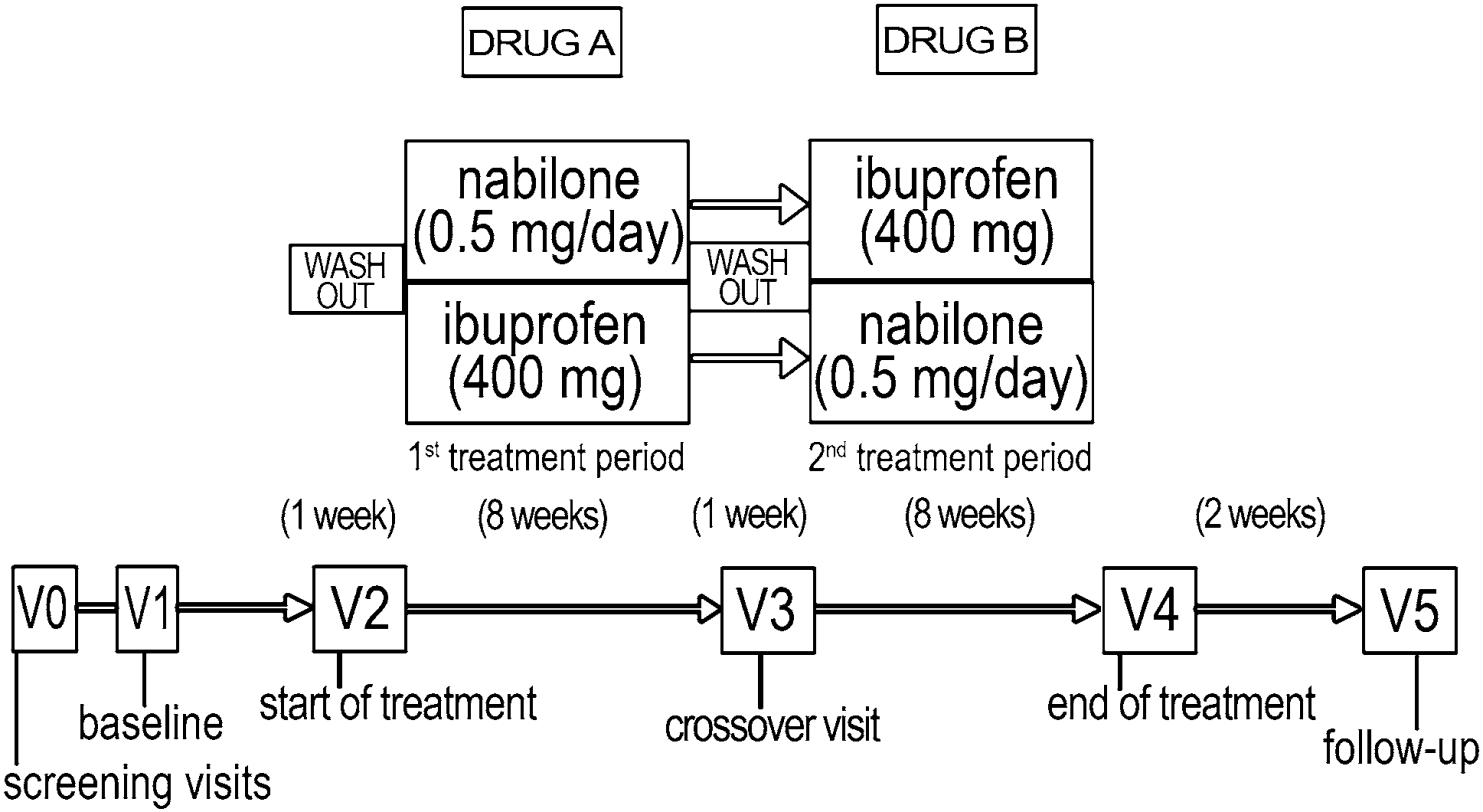
Study design

The study lasted 20 weeks and the protocol consisted of six visits. V0: screening visit; V1: enrolment visit (baseline); V2: dispensing the Drug A container (60 capsules) and start of the first period of treatment (after 1 week from discontinuation of the overused medications at the day hospital of the headache centre); V3: crossover visit, dispensing Drug B container (60 capsules) and start of the second period of treatment (after 1 week of washout); V4: end of treatment visit; V5: follow-up visit, 2 weeks after discontinuation of treatment (Fig. 1).

At each scheduled visit (V1–V5), subjects enrolled in the study, who had given their written informed consent, were examined (particularly as regards the evaluation of headache characteristics) and their vital signs and details of any concomitant medication were recorded. Specifically, a detailed medication history was recorded, including prior prophylactic and symptomatic treatments (the type of drugs used, length of use and any adverse event requiring discontinuation).

In addition, at each visit, the headache diary was reviewed and self-assessment tests were administered to patients.

### Outcome measures

The main aim of the study was to evaluate the efficacy and tolerability of nabilone (0.5 mg/day) for the treatment of MOH.

Primary outcomes to assess the efficacy of treatment were the reduction of the headache frequency, the duration and intensity of headache pain and the amount of daily analgesic consumption. Headache frequency was evaluated using the Headache Index (HI), i.e. the number of headache days per month. The mean duration of pain was evaluated calculating the hours of pain per day (reported in the headache diary); the mean intensity of pain was recorded using the 10 cm Visual Analogue Scale (VAS), which was administered at each visit. In addition, the reduction in the number of analgesics or antimigraine drugs taken during the observation period, was considered as an indirect efficacy parameter and it was measured as daily analgesic intake (DAI).

The secondary outcome measures were the improvement in the quality of life and mental health, assessed through the administered: HIT-6™ (Headache Impact Test), SF-36 questionnaire and the Zung Depression and Anxiety Scales. We also recorded the level of dependence using the Leeds Dependence Questionnaire (LDQ) appropriately modified for headache and consumption of analgesics [28]. This scale does not indicate whether consumption is of a risky level and it was used to monitor changes during the various phases of the study (0 = no dependence; 1–10 = low to moderate dependence; 11–20 = moderate to high dependence; 21–30 = high dependence) [29].

### Safety

The safety and tolerability of the drugs were evaluated at V2, V3, V4 and V5. The safety was assessed by measuring the blood pressure, heart rate and through a medical examination during which the patients were asked about any adverse events during the study period. Moreover, by administration of a diary in which patients were asked to record any adverse events occurring during the treatments and the follow-up period.

### Statistical analysis

The continuous variables were expressed as mean ± standard deviation. To compare all the clinical outcomes (primary and secondary) between the different treatment periods, we used the *t* test for paired data. To compare clinical outcomes between single and multi drug overuser, we used *t* test for unpaired data. All the tests were two-tailed and *p* < 0.05 was considered statistically significant. STATA software (version 10, StataCorp LP, TX, USA) was used for the statistical analyses.

### Study oversight

The corresponding author prepared the first draft of the manuscript and decided to submit the manuscript for publication, after which all the authors worked together to edit the subsequent drafts. All the authors examined and approved the final draft of the manuscript and assumed responsibility for the accuracy and completeness of the data and data analysis and the consistency between the study and the trial protocol.

## Results

Thirty MOH patients aged between 35 and 65 years (mean ± SD = 52.7 ± 9.6), were recruited after the screening visit and allocated according to the randomization schedule; 20 females (66.6 % aged 53.3 ± 9.1 years) and 10 males (33.3 % aged 49.7 ± 11.8 years). All subjects had suffered from chronic headache for at last 3 years. Mean duration was similar for both men and women: 10.3 ± 10.7 and 13.6 ± 10.8 years, respectively. All subjects had a current history of overuse of analgesics or antimigraine drugs; for this study we took into account drugs overused for the 3 months prior to the start of the trial. Medication overused involved triptans in 53 % of the subjects enrolled, combination medications (CM) in 37 % and NSAIDs in 30 %.

Twenty-six subjects completed the study and four dropped out after randomization and allocation. In two cases patients stopped the treatment due to the side effects of the medication (one for nabilone and one for ibuprofen). Other two subjects interrupted the study spontaneously: one subject simply changed his mind after completing the initial washout period, without starting the trial treatment (dropout at V2), and the other one dropped out due to lack of efficacy during the first treatment period (at V3).

**Fig. 2:**
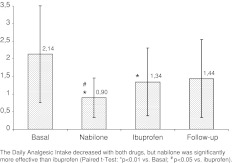
DAI during the trial

At baseline, on average, the patients enrolled had a high analgesic intake (DAI 2.1 ± 1.4) and a high level of drug dependence (15.9 ± 6.3), according to the LDQ score (Fig. 2).

The highest DAI values were observed in those subjects in whom CM (2.8 ± 1.67) was the main overused drug, followed by NSAIDs (1.7 ± 1.03) and then triptans (1.6 ± 0.69), without statistical significant differences between the drugs.

The efficacy data for the main indicators considered are given in Table 1 and the quality of life data is summarised in Table 2.

**Table 1:** Efficacy data of primary outcomes

	Basal	Nabilone	Ibuprofen	Follow-up
*n*	30	26	26	26
HI	0.95 ± 0.1	0.72 ± 0.3**	0.78 ± 0.3**	0.77 ± 0.3*
DAI	2.1 ± 1.4	0.89 ± 0.5**^,∘^	1.34 ± 0.9**	1.44 ± 1.1**
VAS	7.9 ± 1.6	5.7 ± 1.9**^,∘^	6.6 ± 2.2**	6.2 ± 2.4**
LP (h)	16.1 ± 7.1	8.7 ± 6.6**	10.4 ± 7.3*	11.1 ± 7.6**
LDQ	15.9 ± 6.3	9.2 ± 5.9**^,∘^	13.8 ± 6.6	11.9 ± 6.1*
PFD	2.1 ± 0.2	8.1 ± 9.3*	6.6 ± 6.3*	6.9 ± 6.3*

Both drugs showed improvements in all outcomes, but nabilone was always more effective than ibuprofen, with statistically significant differences in DAI, VAS and LDQ values

*HI* Headache Index, *DAI* daily analgesic intake, *VAS* Visual Analogue Scale, *LP* lasting of pain, *LDQ* Leeds Dependence Questionnaire, *PFD* pain free days/month

Paired *t* test * p < 0.05 and ** p < 0.01 versus basal; ^∘^ *p* < 0.05 versus ibuprofen

**Table 2:** Evaluation of the quality of life

	Basal	Nabilone	Ibuprofen	Follow-up
HIT-6	67.3 ± 5.2	62.8 ± 8*	64.9 ± 9.5	63 ± 8.7
SF-36 mental	35.4 ± 11.7	40.2 ± 10.4*	38.8 ± 11.1	40.6 ± 15.9
SF-36 physical	33.1 ± 8	39.5 ± 7.7*	37.2 ± 8.1	38 ± 9.8
ZAS	41.3 ± 7.8	37.9 ± 11.5	39.2 ± 9.5	40.5 ± 11.7
ZDS	44.1 ± 9.3	41.3 ± 11.1	41.3 ± 9.2	43.2 ± 12.7

The improvements recorded in quality of life scales occurred only with nabilone

HIT-6™ Headache Impact Test, *SF-36* Short Form Health Survey, *ZAS* Zung Anxiety Scale, *ZDS* Zung Depression Scale

Paired *t* test * * p* < 0.05 versus basal

Both drugs showed improvements compared to baseline in all the primary endpoints, however, certain differences were observed between the two treatments. Nabilone was directly superior to ibuprofen in reducing DAI, pain intensity and the level of dependence (Table 1; Fig. 2). In addition, the quality of life indicators changed with nabilone, but not with ibuprofen: a significant improvement was seen in SF-36 Scale (for both physical and mental components) and HIT-6™ Scales (Table 2).

A deeper analysis showed that the improvements compared to baseline recorded with ibuprofen only occurred in subjects taking ibuprofen during the first period of treatment (i.e. DRUG A), but not in subjects taking ibuprofen during the second period of treatment (i.e. DRUG B). The improvements recorded with nabilone compared to baseline, instead, took place regardless of when the therapy was received.

As far as the post-treatment results are concerned (recorded at the follow-up visit, 2 weeks after discontinuation of the DRUG B), the improvements compared to baseline persisted. However, these improvements depended on the sequence of the pharmacological treatments since, compared to baseline, only patients receiving nabilone during the last 2 months maintained a significant prolonged improvement (carry-over effect), in the HI, DAI, VAS and HIT-6™ indices. The subjects who received ibuprofen during the last 2 months of treatment, on the other hand, did not show any improvement compared to baseline. The post-treatment results are given in Table 3.

**Table 3:** Post-treatment outcomes

	Ibuprofen		Nabilone	
	Basal	Follow-up	Basal	Follow-up
HI	0.97 ± 0.1	0.86 ± 0.3	0.93 ± 0.1	0.65 ± 0.4*
DAI	2.02 ± 1.2	1.8 ± 1.3	2.34 ± 1.6	0.99 ± 0.8*
VAS	8 ± 1.6	6.75 ± 2.4	7.8 ± 1.7	5.55 ± 2.5**
HIT-6™	68.4 ± 5.7	63.1 ± 11.9	66.7 ± 4.8	64 ± 6.8*

The post-treatment improvements occurred only in patients who received nabilone during the second period of treatment

*HI* Headache Index, *DAI* daily analgesic intake, *VAS* Visual Analogue Scale; HIT-6™ Headache Impact Test

Paired *t* test * *p* < 0.05 and ** *p* < 0.01 versus basal

With regard to the habits of taking the overused medications, there were two types of patients: those who were overusing just one medication, who were termed single drug overusers (SDO 15 subjects) and those who were overusing two or more different medications, who were termed multi drug overusers (MDO 15 subjects).

**Fig. 3:**
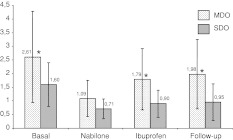
Time-course DAI in multidrug overusers versus single drug overusers

The MDO group had far higher DAI values than the SDO group: 2.61 ± 1.6 versus 1.60 ± 0.79, respectively (*p* < 0.05). This difference in the consumption rate between the two groups of overusers persisted at each visit. We always observed higher DAI values in the MDO group (*p* < 0.05), however, this did not occur during the period of treatment with nabilone. During the treatment with nabilone, but not with ibuprofen, the DAI dropped regardless of the type of overuse, for both SDO and MDO patients (Fig. 3).

### Safety

All the adverse events (Table 4) were of a mild intensity and disappeared after discontinuation of the medication or spontaneously after a few days of treatment. The main AEs, which caused the withdrawal of two patients, were of a moderate intensity in both cases. One woman reported mild gastric discomfort during treatment with ibuprofen, whereas during treatment with nabilone, one man complained of mild cognitive disorders (loss of concentration and memory), symptoms that disappeared within a month after withdrawal. Throughout the entire study, there were no changes in blood pressure, heart rate or body weight.

**Table 4:** Adverse Events

	Nabilone	Ibuprofen
Dizziness	2	–
Sleep disorders	–	1
Decreased appetite	1	2
Vomiting	2	–
Nausea	1	2
Drowsiness	–	–
Asthenia	2	–
Epigastric discomfort	1	2
Dry mouth	2	
Loss of attention	–	1
Memory impairment	–	–
TOTAL	11	8

Adverse events were mild and disappeared after few days of treatment or after drug discontinuation

## Discussion

Cannabinoids, like many analgesics and recreational drugs, act on the brain’s reward pathways. Cannabinoid-1 receptors (CB1R) are co-localized with the opioid receptors on the dopaminergic cells of the nucleus accumbens, probably the most important structure in human reward pathways, which partly overlaps the antinociceptive pathways [30–32].

The oral administration of cannabinoid drugs shows poorer bioavailability than when these drugs are administered by inhalation. An oromucosal spray of THC was one way of releasing active principle into the central nervous system, however, the rapid administration of cannabinoid drugs had different effects to those observed with slow absorption: the reward system is activated by a rapid rise in cannabinoids concentration, such as to obtain a significant euphoric effect (a ‘high’), the main cause of dependence. The oral cannabinoids administration, on the other hand, avoids concentration peaks and with chronic administration, the individual differences in bioavailability are overcome [23, 33]. The use of nabilone, a cannabinoid1-receptor agonist, would therefore appear reasonable in the treatment of MOH for which central mechanisms are hypothesised in the maintenance of chronic head pain due to medication overuse.

We studied a group of patients who had been suffering from MOH for a long time (on average more than 12 years) and who had used various pharmacological and other approaches, without achieving any positive results.

When resistant to conventional medical treatment and prophylactic medication this condition is known as refractory chronic migraine [34].

Our patients presented almost daily headache (HI = 0.95 ± 0.1), with really high DAI values at baseline (2.1 ± 1.4). In these patients, the main unresolved problem is the excessive use of drugs for the acute treatment and the overall deterioration experienced in their quality of life, so the headache symptoms should be considered as part of the issue as a whole [2, 6, 7].

Nabilone seemed more helpful in reducing the intensity of pain than the frequency. The mean intensity of pain (measured using the VAS) dropped significantly (*p* < 0.01) both with nabilone (−27.9 %) and with ibuprofen (−17.8 %), with a difference between the two treatments in favour of nabilone (*p* < 0.05).

The frequency of headache had only very small improvements, probably due to the short period of treatment.

Nabilone showed a remarkable improvement in drug consumption. So, the most important effect recorded with nabilone was especially in reducing drugs overuse, with DAI values more than halved the baseline. DAI dropped during both treatments: −36.2 % with ibuprofen (*p* < 0.01) and −57.7 % with nabilone (*p* < 0.01), which was significantly superior to ibuprofen, in reducing analgesic intake (DAI = 0.89 ± 0.5 and 1.34 ± 0.9, respectively, *p* < 0.05). In addition, a deeper analyses showed significant differences between the DAI in single drug overusers and in multi drug overusers: during treatment with nabilone both SDO and MDO improved in a similar way, however, this was not so for the period of treatment with ibuprofen as MDO patients maintained higher overuse than SDO (Fig. 3). This result agrees with the clinical observation that multi drug overusers experience greater difficulties in reducing DAI and are less sensitive to treatments. Nabilone seems able to help patients with multi drug overuse [12].

This great reduction in DAI recorded with nabilone is also concordant with the changes in the consumption habits of drugs, recorded by the Leeds Dependence Questionnaire. The LDQ score showed a high baseline value, of about 16 points and was similar to the score obtained in a previous study on patients suffering from chronic daily headaches [28]. The questionnaire indicated a significant reduction in the level of dependency compared to baseline during treatment with nabilone (−42.2 %; *p* < 0.01), but not with ibuprofen (Table 1).

A reduced use of medication implies a reduced effect of headache pain on the quality of life. The slight improvements in the quality of life (in HIT-6™ and SF-36), were recorded only with nabilone and not with ibuprofen (Table 3); the small degree of these improvements is probably in relation to the short duration of treatment.

Nabilone’s ability to reduce DAI in both types of overusers (SDO and MDO) associated with a reduction in the LDQ score suggests that nabilone could affect the degree of dependence in both of these conditions.

The main limits of our research were the small sample size and the short duration of the study. However, our results were obtained in a selected chronic headache population considered a representative sample of the most severe MOH patients who failed to respond to all available pharmacological treatments.

## Conclusions

To conclude, nabilone, a cannabinoid 1-receptor agonist, at daily doses, would appear beneficial for patients suffering from MOH, primarily in reducing the intensity of pain and the analgesic intake and appeared to be significantly more efficacious than ibuprofen. In addition, nabilone alone reduced the level of drug dependence (LDQ −41 %, *p* < 0.01) and improved the quality of life scales (*p* < 0.05). The number of days with headache was not significantly reduced in the same way as the other indicators, probably due to the short duration of the study. Side effects were infrequent, of mild intensity and disappeared after discontinuation of the treatment. This randomized, controlled trial evaluated the benefits of nabilone on headache, analgesic consumption and the improvements in quality of life in patients with MOH. Nabilone would also appear to be safe. Larger-Scale studies are required to confirm the effectiveness and safety of nabilone [1, 7].

What is already known about this study: although cannabinoids have been used as painkillers for centuries, there is little evidence-based information available on their use. At low doses, they have few psychotropic side effects, which disappear rapidly in patients with chronic headache.

What this study adds: nabilone, a synthetic oral cannabinoid, is efficacious in the treatment of medication overuse headache.
